# Metagenomic Insights into the Carbohydrate-Active Enzymes Carried by the Microorganisms Adhering to Solid Digesta in the Rumen of Cows

**DOI:** 10.1371/journal.pone.0078507

**Published:** 2013-11-05

**Authors:** Lingling Wang, Ayat Hatem, Umit V. Catalyurek, Mark Morrison, Zhongtang Yu

**Affiliations:** 1 Department of Animal Sciences, The Ohio State University, Columbus, Ohio, United States of America; 2 Department of Biomedical Informatics, The Ohio State University, Columbus, Ohio, United States of America; 3 Department of Electrical and Computer Engineering, The Ohio State University, Columbus, Ohio, United States of America; 4 CSIRO Livestock Industries, St Lucia, QLD, Australia; Wageningen University, The Netherlands

## Abstract

The ruminal microbial community is a unique source of enzymes that underpin the conversion of cellulosic biomass. In this study, the microbial consortia adherent on solid digesta in the rumen of Jersey cattle were subjected to an activity-based metagenomic study to explore the genetic diversity of carbohydrolytic enzymes in Jersey cows, with a particular focus on cellulases and xylanases. Pyrosequencing and bioinformatic analyses of 120 carbohydrate-active fosmids identified genes encoding 575 putative Carbohydrate-Active Enzymes (CAZymes) and proteins putatively related to transcriptional regulation, transporters, and signal transduction coupled with polysaccharide degradation and metabolism. Most of these genes shared little similarity to sequences archived in databases. Genes that were predicted to encode glycoside hydrolases (GH) involved in xylan and cellulose hydrolysis (e.g., GH3, 5, 9, 10, 39 and 43) were well represented. A new subfamily (S-8) of GH5 was identified from contigs assigned to *Firmicutes*. These subfamilies of GH5 proteins also showed significant phylum-dependent distribution. A number of polysaccharide utilization loci (PULs) were found, and two of them contained genes encoding Sus-like proteins and cellulases that have not been reported in previous metagenomic studies of samples from the rumens of cows or other herbivores. Comparison with the large metagenomic datasets previously reported of other ruminant species (or cattle breeds) and wallabies showed that the rumen microbiome of Jersey cows might contain differing CAZymes. Future studies are needed to further explore how host genetics and diets affect the diversity and distribution of CAZymes and utilization of plant cell wall materials.

## Introduction

The rumen microbiome is among the most rapid and efficient in degrading cellulose and hemicellulose. Even so, up to half of the ingested cellulosic material can pass the rumen undigested due to both its inherent recalcitrance and the short retention time in the rumen [1]. Hydrolysis of cellulosic biomass to monomeric sugars is also the bottleneck step to producing cellulosic biofuel [2]. Therefore, the rumen microbiome has attracted enormous interest in the pursuit of improved feed utilization efficiency of the livestock industry and cost-competitive production of cellulosic biofuel [3]–[7]. Because most of the glycoside hydrolysis occurs in the biofilm adhering to the ingested feed particles [8], most metagenomic studies focused on the bacterial community adherent to the fiber particles [3]–[6]. To date several large metagenomic studies have reported the diversity of carbohydrate-active enzymes (CAZymes) from the rumen microbiome of Angus beef cattle [4], Guernsey dairy cattle [3], reindeer [5], and yak [7]. Pope *et al* [9] also reported new CAZymes and polysaccharide utilization loci (PUL)-like systems that contain genes encoding β-1,4-endoglucanases and β-1,4-endoxylanases in the foregut microbiome of tammar wallaby native to South and Western Australia. However, the CAZyme diversity in the rumen of dairy cattle has not been explored extensively even though Hess *et al*. [3] has investigated the CAZymes of bacteria adhering to switchgrass (a bioenergy crop placed in nylon bags) in the rumen of two Guernsey dairy cows.

Studies on phylogenetic diversity and community structure based on 16S rRNA genes have documented that the rumen microbiome can be shaped by both diet and host genetics. The effects of host genetics on the rumen microbiome were demonstrated by some unique features of the rumen microbiome of African ruminant species [10], reindeer [11], yak [12], buffalo [13], and comparative studies among different species [14], while dietary effects were revealed by the shifts of microbial populations upon changes in diets [15], [16]. Although the diversity of CAZy genes in the rumen of Angus, Guernsey, buffalo, reindeer, and yak have been explored, no study has been reported for Jersey cattle, one of the major dairy cattle breeds in the US and other countries. The objectives of this study were to explore the diversity of CAZy genes in the rumen of Jersey cattle and to compare it with that of the CAZy genes found in the rumen of other species or breeds. Instead of shotgun metagenomic sequencing, which generates large numbers of gene fragments that can lead to diversity overestimate and incorrect annotation, we focused on sequencing and bioinformatic analysis of 120 fosmid clones that expressed hydrolase activities towards polysaccharides (cellulose and xylan). New putative CAZy genes of full length were found and some features of gene organization suggest synchronized polysaccharide utilization with other metabolism.

## Results and Discussion

Metagenomics has been used to explore the diversity of CAZymes in the rumen or gut of herbivore animals. This is, however, the first metagenomic study that used an activity-based fosmid library in examining the CAZymes diversity of the biofilm colonizing feed particles, where fiber digestion takes place [8], in the rumen of Jersey cows. From screening 14,000 fosmid clones (30 – 40 kb insert per clone, corresponding to 0.42 – 0.56 Gb of rumen metagenomic DNA in total), 120 clones were found hydrolase-active toward cellulose (34 clones), xylan (52 clones), starch (1 clone), and esculin hydrate (33 clones). These fosmid clones were pyrosequenced as a pool, yielding > 600,000 high-quality reads with an average length of 455 bp (about 280 Mb DNA sequences in total), which represent an average sequencing coverage of 58× for the sequenced fosmids. A two-staged integrated sequence assembly approach using multiple software tools (see **Materials and Methods**) generated 66 contigs of full-length inserts and 246 contigs of partial inserts longer than 2 kb (Table S1). Inter-contig comparison showed that only four contigs had similar sequences: Contig785_n_1807_cl (9.1 kb, GenBank accession number: KC246876) and Contigcl_48 (20 kb, KC246849) shared 99% sequence similarity over 92% of the shorter contig, and Contig1490 (6.2 kb, KC247041) and Contig1831 (36.6 kb, KC246871) shared 93% sequence similarity over 75% of the shorter contig. All the other contigs shared little sequence similarity, suggesting that most of the 120 fosmid clones were derived from either different organisms or different chromosomal fragments of the same organism.

In total, 3,553 open reading frames (ORFs) were predicted, and most of them have a starting codon (i.e., ATG) and a universal stop codon (i.e., TAA, TGA or TGA), suggesting that most of the ORFs probably represent full-length genes. The average length of all the predicted proteins was 347 amino acids, with a range from 19 to 3655 amino acids. To reflect the bioinformatic nature of the analysis, all the predicted ORFs with a putative predicted function were considered putative genes. Unlike shotgun sequencing that often produces a lot of gene fragments (e.g. 45% in the study by Hess [3]), targeted sequencing of fosmids expressing activities of particular interest produces more full-length genes. This has enabled detection of signal peptides in a relatively high proportion (16.5%) of the CAZy genes predicted. Conceivably, the full gene length probably also improved annotation accuracy and avoided artificial inflation of diversity estimates.

### Taxonomic Assignment of the Metagenomic Contigs and ORFs

No marker genes such as 16S rRNA genes were identified in this study. Thus, the taxonomic origins of 107 long contigs (> 10 Kb) were predicted using MEGAN 4 and PhyloPythiaS to infer the probable host organisms (Table S2). MEGAN could only assign most of the contigs to phylum level. PhyloPythiaS assigned most of the contigs to the same phyla as MEGAN, except assigning 8 contigs predicted to be *Firmicutes* by MEGAN to *Actinobacteria*. PhyloPythiaS assigned 12 of the 21 contigs that could not be assigned to any phyla by MEGAN to *Firmicutes*. PhyloPythiaS assigned most of the contigs to a deeper lineage than MEGAN, but not to existing species (Table S2). Besides, PhyloPythiaS assigned all except one of the *Firmicutes* contigs to the order *Selenomonadales*. The taxonomic assignments by PhyloPythiaS may be biased towards *Selenomonadales* due to the lack of a good training dataset [17]. Nevertheless, the inability to assign nearly all of the contigs to known genera or species indicates that most of them might have been derived from so far uncultured species or cultured species whose genome has not been sequenced. The results also corroborate a previous report that compared with MEGAN, PhyloPythiaS is more sensitive to unknown organisms [18].

**Figure 1:**
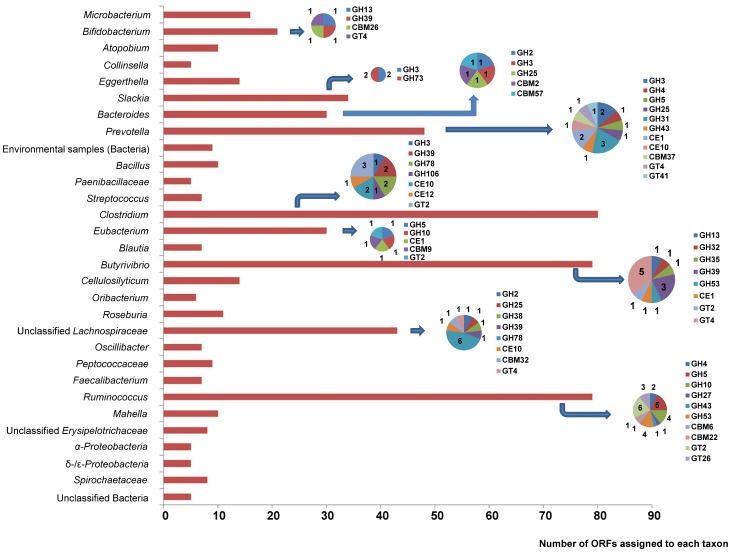
Distribution of CAZy families at the lowest taxon assigned by MEGAN. The taxa were based on the NCBI taxonomy. The numbers of CAZy genes identified in each taxon were shown in pie chart. CBM, carbohydrate binding module, GH, glycoside hydrolase, GT, glycosyl transferase, CE, carbohydrate esterase, PL, polysaccharide lyase. Environmental samples (Bacteria), bacteria recovered from environment that could not be assigned to existing phyla; Unclassified bacteria, bacteria that could not be classified to existing phyla.

The host organisms of all the ORFs were also inferred using MEGAN. Overall, 1256 of the 3553 predicted ORFs were assigned to the phylum *Firmicutes*. Of all the 3553 predicted ORFs, only 622 were assigned to 21 genera or other sub-phylum taxa (Figure 1). The Major genera included *Clostridium* (2.2% of total ORFs), *Ruminococcus* (2.2%), *Butyrivibrio* (2.2%), *Prevotella* (1.4%), and unclassified *Lachnospiraceae* (1.2%). A greater number of GH genes (17 in total) were assigned to *Ruminococcus* than to other genera. The other major genera were also represented with multiple CAZy genes. Given the high abundance of *Prevotella* in the rumen microbiome [19]–[21], it is intriguing that it was not well represented by the identified CAZy genes. It should be noted that although many CAZy genes (67 CAZy genes including 39 GHs) were assigned to known fibrolytic genera (e.g., *Clostridium*, *Ruminococcus*, *Butyrivibrio*, and *Prevotella*), none of them appeared to be derived from the type species or type strains of these genera, indicating a greater diversity of cellulolytic bacteria than that represented by those type species. The assignment of many CAZy genes to the above genera may reflect the presence of conserved functional domains shared among fibrolytic bacteria.

### Prediction of Gene Functions

**Figure 2:**
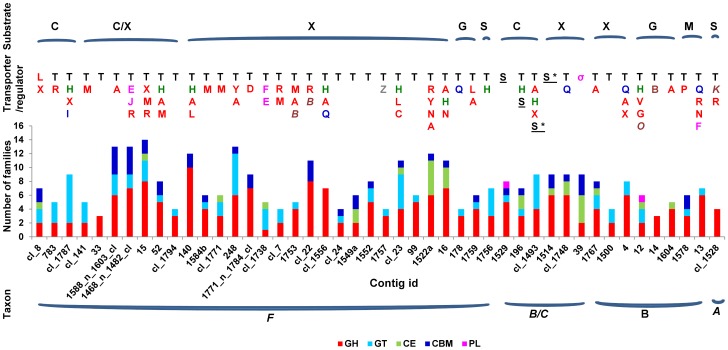
Distribution of CAZy families and genetic features in the contigs that contain > 2 degradative CAZy families with clear substrates designated. GH  =  glycoside hydrolase, GT  =  glycoside transferase, CE  =  carbohydrate esterase, CBM  =  carbohydrate binding module, PL  =  polysaccharide lyase. Taxa: *F*  =  *Firmicutes*, *B/C  =  Bacteroidetes/Chlroobi*, *B*  =  Bacteria, *A  =  Actinobacteria*. Substrate: C  =  cellulose, X  =  xylan/hemicellulose, G =  Glucosides, S  =  starch or sucrose, M  =  Mannan. T (black)  =  transporter. H (green)  =  sensor histidine kinase. Transcriptional regulators (red capital letters): R  =  transcriptional regulator, L  =  LacI family, X  =  XRE family, M  =  MerR family, A  =  AraC family, Y  =  LysR family, D  =  DeoR family, C  =  Cro/CI family, N  =  TetR, V  =  VicR, G  =  GntR family, P  =  PadR family. Mobile elements (blue): I  =  integrase, Q  =  transposase. Sigma factor and related genes (pink): E  =  ECFσ, F  =  anti-sigma factor antagonist, J  =  anti-YlaC sigma factor, σ  =  sigma-70 factor. Sugar related regulatory factors (italic brown): B  =  sugar fermentation stimulation protein SfsA, O  =  sucrose operon repressor ScrR, K  =  trehalose operon repressor. Sus-like system genes (underlined): S  =  SusC/SusD, S*  =  SusC. Z (grey)  =  zinc finger domain protein of LSD1 subclass.

The predicted ORFs were annotated by comparison with several databases. Comparison to the GenBank NR Protein databases revealed 3,123 genes (88% of the total predicted ORFs) that were homologous to those deposited in that database (E value < 1e-5), with an average sequence similarity being about 64.2%. Only about 17% of the predicted ORFs were identical and 22% were > 90% similar to those in the NR database over the aligned regions. Further comparison to the COG database revealed 20 categories of genes (Table S3). A high percentage of the genes were predicted to be involved in carbohydrate transport and metabolism (11.1% of the total ORFs). This is expected given the source of the samples (biofilm adhering to solid digesta) and the targeted sequencing of only the carbohydrase-active fosmid clones. Many genes putatively associated with transcription (category K, 5.3% of total ORFs), signal transduction (category T, 3.8%), amino acid transport and metabolism (category E, 6.2%), and protein synthesis (category J, 3.9%) were found in proximity of carbohydrate-metabolizing genes (Table S4, Figure 2). It remains to be determined if such a gene organization reflect synchronization between carbohydrate degradation/utilization and other metabolic processes such as protein synthesis.

Based on analysis using the KEGG database, 341 genes, which accounted for nearly half of the metabolic genes predicted, were probably involved in carbohydrate metabolism (Figure S1A). Among them, 134 were predicted to be involved in starch and sucrose (Figure S1B), which is a metabolism category for not only starch and sucrose, but also a number of other carbohydrates, including glycogen, maltose, cellobiose, trehalose, glucose, fructose, xylose, glucuronate, mannose, rhamnulose, and arabinose. Because many of these saccharides are the hydrolytic products of the major feed components (cellulose, xylan, and starch) of cattle, the rich representation of this gene category is not surprising. Additionally, some of the genes were classified into categories involved in the major metabolic pathways of butanoate, citrate cycle, glyoxylate and dicarboxylate, pentose and glucuronate interconversions, propanoate, and pyruvate (data not shown). These findings suggest concentrated arrangement of metabolic genes involved in the major steps of polysaccharide metabolism, such as polysaccharide depolymerization, transport, fermentation, and regulation, on the genome of fibrolytic microbes. From an evolutionary perspective, such arrangements may enable the host bacteria to efficiently utilize the nutrients obtained from recalcitrant substrates [22].

### Plant Carbohydrate-Active Enzymes

Comparison of the predicted ORFs with the CAZy database uncovered 575 (16% of the total ORFs) unique CAZy genes (E value < 1e-5) within 154 contigs (Table S5). The numbers of the predicted CAZy genes in these contigs varied from 1 to 12, with 39 contigs containing 6 or more CAZy genes, suggesting varied arrangement of CAZy genes. At least 120 of the CAZymes could be functional because they originated from the 120 fosmids that expressed glycoside hydrolase (GH) activity during the activity screening. Most (56.2%) of the CAZy genes were assigned to GH families (42 families in total) and the remaining genes were assigned to other CAZy families, CBM, GT, CE and PL. (Tables 1, Table S5). Putative arabinogalactan endo1,4-beta-galactosidase (GH53) and α-L-rhamnosidase (GH78) gene were also found, likely recovered from bacteria capable of degrading the hemicellulose side chains and pectin. The large number of CAZy genes found is indicative of the potential of various rumen bacteria to utilize carbohydrates as their main substrates.

**Table 1:** Comparison of the major GH families identified in large metagenomes of herbivores.

GH family	Major activity	Macropod^a^	Angus cows-pooled liquid^b^	Angus cows-fiber adherent^b^	Guernsey cows^c^	Reindeer^d^	Yak rumen^e#^	Jersey cows (this study)
Cellulases								
GH5	cellulases	10	7	7	1451	287	1302(12)	24
GH6	endoglucanase	0	NR	NR	0	0	0(0)	1
GH7	endoglucanase	0	NR	NR	1	0	0(0)	0
GH9	endoglucanase	0	7	6	795	109	767(7)	8
GH44	endoglucanase	0	0	0	99	5	0(0)	0
GH45	endoglucanase	0	NR	NR	115	0	13(1)	0
GH48	cellobiohydrolases	0	0	1	3	5	32(0)	0
Total		10(4%)*			2,464(13%)	406(8%)	2114(20)	33(14%)
Endo-hemicellulases								
GH8	endoxylanase	1	8	2	329	35	174(6)	0
GH10	endo-1,4-β-xylanases	11	10	5	1025	190	2664(14)	35
GH11	xylanases	0	2	1	165	8	244(0)	0
GH12	xyloglucanases	0	NR	NR	0	0	0(0)	1
GH26	β-mannanase & xylanases	5	2	5	369	153	537(9)	1
GH28	galacturonases	2	9	3	472	120	244(4)	0
GH53	endo-1,4-β-galactanases	9	15	17	483	125	1066(5)	18
Total		28(10%)			2,843(15%)	631(12%)	4929(38)	55(24%)
Xyloglucanases								
GH16	xyloglucanases	4	0	1	483	116	563(2)	0
GH74	xyloglucanases	1	0	0	385	44	0(2)	0
Total		5(2%)			868(5%)	160(3%)	563(4)	0(0%)
Debranching enzymes								
GH51	α-L-arabinofuranosidases	12	73	61	1249	488	0(9)	1
GH54	α-L-arabinofuranosidases	0	0	1	76	23	111(1)	0
GH62	α-L-arabinofuranosidases	0	NR	NR	1	0	0(0)	0
GH67	α-glucuronidases	5	NR	NR	120	74	1090(2)	0
GH78	α-L-rhamnosidases	25	41	31	1260	313	426(7)	13
Total		42(15%)			2,706(15%)	898(17%)	1627(19)	14(6%)
Oligosaccharide degrading enzymes								
GH1	β-glucosidases	61	7	10	253	122	331(1)	10
GH2	β-galactosidases	24	218	176	1,436	716	942(13)	16
GH3	β-glucosidases	72	207	166	2,844	844	5448(24)	48
GH29	α-L-fucosidases	2	31	26	939	268	899(1)	3
GH35	β-galactosidases	3	21	9	158	39	468(0)	2
GH38	α-mannosidases	3	22	15	272	116	90(1)	1
GH39	β-xylosidases	1	2	2	315	76	159(1)	18
GH42	β-galactosidases	8	10	12	374	95	207(4)	0
GH43	arabino/xylosidases	10	68	59	2932	787	2313(28)	28
GH52	β-xylosidases	0	0	0	1	2	0(0)	0
Total		184(70%)			9,524(52%)	3,065(60%)	10857(73)	126(55%)
Other domains associated with GHs								
Cohesion		0	0		80	52	51(0)	0
Dockerin		41	8		188	92	516(0)	2
SusC		36	9		3110	1122	NR	4
SusD		42	11		1889	685	NR	3
Metagenomic size		0.054Gb	0.024Gb	0.027 Gb	268Gb	0.30Gb	9.4Gb (0.3Gb)	0.28Gb

Data are presented using the format described in reference [5]. GHs were grouped based on their major activity in plant fiber degradation.

NR, not reported.

a Pope *et al*, 2010 [9] (sequencing of activity-screened fosmids using 454 GS FLX).

b Brulc, *et al*, 2009 [4]. The number of fiber-adherent samples was the average of three fiber adherent samples (shotgun metagenomic sequencing using 454 GS20).

c Hess *et al*, 2011 [3] (shotgun metagenomic sequencing using Illumina GAII and HiSeq2000).

d Pope *et al*, 2012 [5] (shotgun metagenomic sequencing and sequencing of activity-screened BAC clones using 454 GS FLX Titanium).

e Dai *et al*, 2012 [7] (sequencing of activity-screened BAC clones using 454 GS FLX Titanium).

* Percentage of each group relative to the total number of GH’s identified in each metagenomic dataset.

# Numbers inside and outside parentheses are those retrieved from the BAC library and shotgun sequencing, respectively. The data inside the parentheses showed the dereplicated GHs.

**Figure 3:**
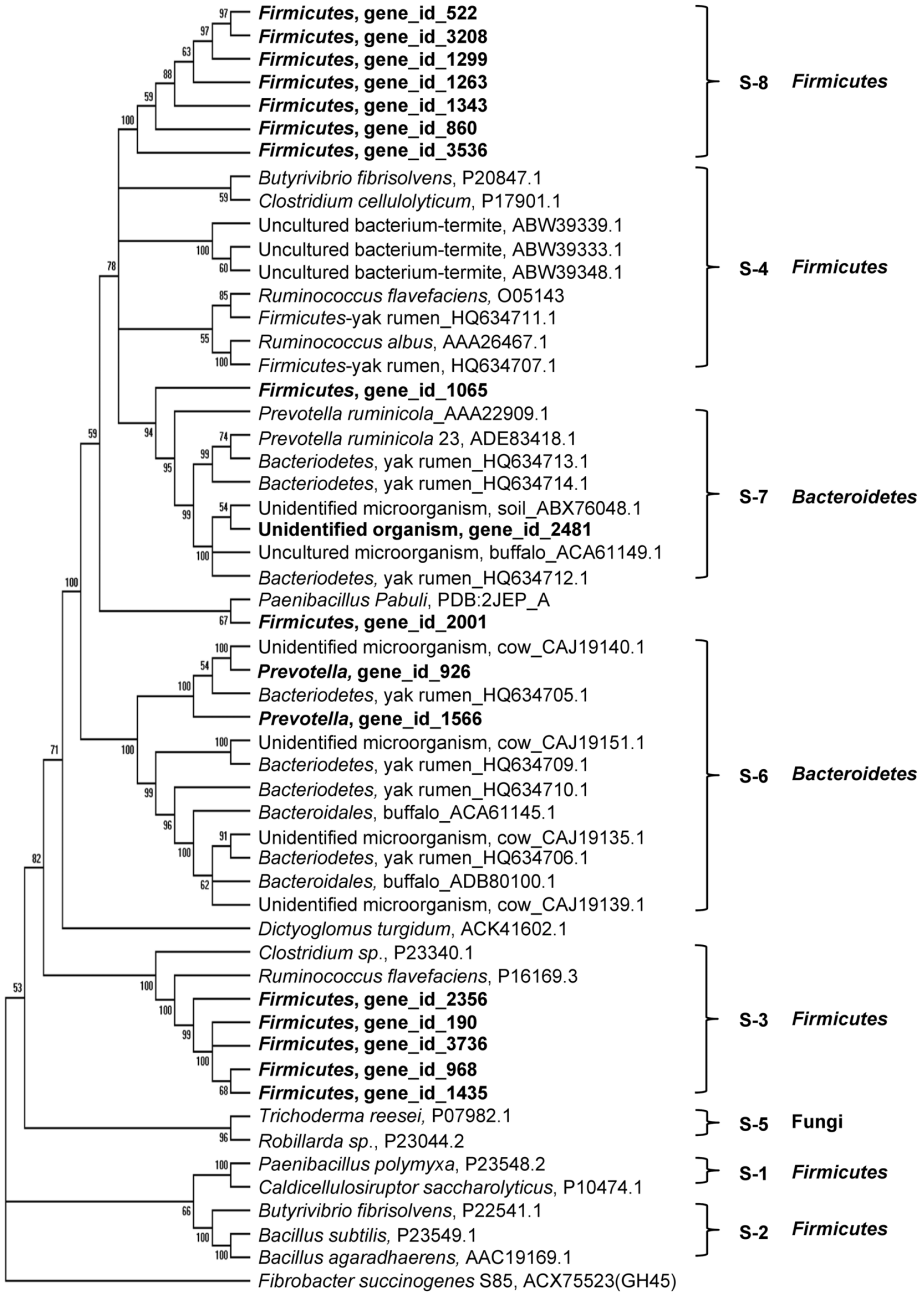
A phylogenetic tree of the putative GH5 proteins from the available metagenomic datasets. The tree was drawn using the Neighbor-Joining method with bootstrap replication of 500. Branches with ≤ 50% bootstrap support were collapsed. The evolutionary distances were calculated using the p-distance method. Names of proteins (or predicted proteins) were presented by the source organisms and GenBank accession number. The predicted proteins identified in this study are bolded. The GH45 protein of *Fibrobacter succinogenes* S85 was used as an outgroup. S-1 to S-8 are subfamilies of GH5 proteins.

In the large metagenomes reported previously [3]–[5], [7], [9] and the present study, GH5 is the most diverse and predominant family of cellulases (Table 1). The GH5 genes have been clustered into seven subfamilies (S-1 to S-7) [7]. GH5 genes belonging to these subfamilies were also identified in the present study (Figure 3). Besides those clustered with the previously identified GH5 subfamilies, seven new GH5 genes formed a new subfamily (referred to as S-8), which consisted of genes assigned to *Firmicutes*. Based on all available data of the GH5 family shown in Figure 3, it is intriguing that the distribution of the GH5 subfamilies showed significant taxonomic bias: Subfamilies S-1 to S-4 and S-8 were mainly assigned to *Firmicutes*, while subfamilies S-6 and S-7 were primarily assigned to *Bacteroidetes*.

More than half of the predicted CAZy genes were located in the 80 contigs that contained several categories of genetic elements previously found in polysaccharide utilization loci (PUL) (Table S4), suggesting that the long contigs (full and nearly full length) allowed for examination of organizational patterns of fibrolytic gene clusters (e.g., PUL) and occurrence of regulatory genes. The occurrences of CAZy genes in the contigs were uneven. Among the 80 contigs, only 9 were shorter than 10 Kb. The elements in the PULs included transcriptional regulators (e.g., families LacI, AraC, MerR, and XRE), nutrient or ion transporters (e.g., ABC transporters and binding-protein-dependent transport systems), environmental sensors (e.g., two component histidine kinase/response regulators), and other proteins involved in sugar metabolism (Figure 2). The AraC family has been reported in other metagenomic studies [7], [9], but not the LacI, MerR, or XRE families. Interestingly also, among contigs assigned to *Firmicutes* the XRE and the MerR families appeared more frequently in the contigs containing cellulase genes, while the MerR family occurred more frequently in the contigs containing xylanase/hemicellulase genes. In addition, some mobile genetic elements, such as insertion sequences (IS) and transposases (Figure 2), were also identified in vicinity of CAZy genes in some of the contigs. This finding may support the possibility of lateral transfer of CAZy genes between rumen microbes and other rumen-inhabiting microorganisms, as previously suggested by Richard and colleagues in a study analyzing the expressed sequence tags of rumen protozoa [23]. One cellobiohydrolase assigned to GH6 was revealed in the present dataset (gene_id 2019, Tables S4, Table S5). This cellobiohydrolase gene was located in a gene cluster containing an integrase catalytic subunit and a transposase, and it had the highest sequence similarity to fungal GH6 cellobiohydrolases when compared to the characterized GH6 available in the CAZy database.

**Figure 4:**
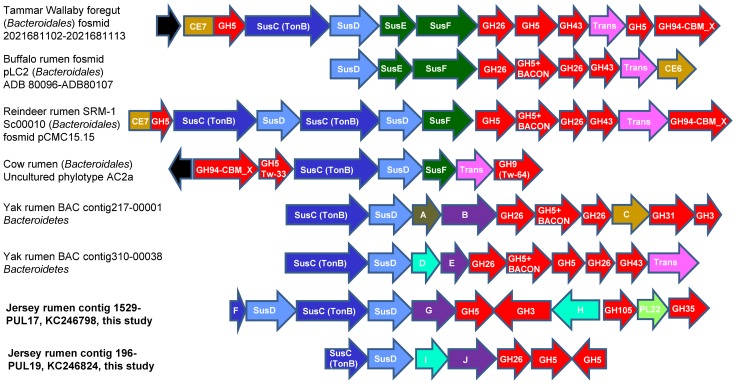
Gene organization of representative cellulolytic PULs identified in available metagenomes and the present study (shown in bold). The genes of the two PULs identified in this study were listed in Table S4. The figure was partly adapted from the paper by Pope *et al* [5]. Consistent with that paper, black genes represent putative response-regulators. SusE/SusF-like genes encode outer-membrane proteins. Trans: putative transporter. BACON: a carbohydrate binding domain. TonB: TonB-dependent receptor family, which functions to transport solutes and macromolecules with a membrane spanning β-barrel proteins. TW-64 and TW-33 are sample IDs for the GH9 and GH5 genes (respectively) identified within the AC2a PUL. GenBank accession numbers and/or IMG Gene Object ID numbers are provided for each cluster/scaffold when available, and details for each gene were listed in Table S6. A: hypothetical lipoprotein; B, E, G, J: putative polygalacturonase; C: sialic acid acetylesterase; D, H, I, hypothetical protein; F: SusC homolog. Both F and the first SusC in contig 196-PUL19 are located at the end of the contigs.

Two of the PULs (Contig1529-PUL17 and Contig196-PUL19), both of which were assigned to *Prevotella* by PhyloPythiaS (Figure 4, Table S4), showed particularly interesting features. Contig1529-PUL17 contained a polygalacturonase gene and a cellulase gene that is highly similar (98.2% identical amino acid sequence) to a functional cellulase previously identified in a small-insert clone library of the rumen microbiome [24], while Contig196-PUL19 contained genes homologous to several different GH genes including polygalacturonase, bi-functional mannanase-xyloglucanase, cellulase, and mannan endo-1,4-beta-mannosidase. The finding of the present study further demonstrated the high frequency of GH43, GH26, GH5, and GH3 in Sus-like clusters and their potential importance in utilizing plant biomass rich in cellulose, xylan and polygalacturonan. The finding of a GH35 and a GH105, both of which have not been reported in PULs, in Contig-PUL17 also suggests that the GH diversity in PULs is probably larger than what has been found, and PULs may be more important in digestion of plant cell wall materials in the rumen than previously suggested [25], [26].

The repertoire of CAZy genes identified in the present study was compared to the sequenced genomes (complete or draft) of the six well studied rumen fibrolytic bacteria (i.e., *Fibrobacter succinogenes* S85, *Prevotella bryantii* B_1_4, *P. ruminicola* 23, *Ruminococcus albus* 7, *R. albus* 8, *R. flavefaciens* FD-1, Table 2) that have been considered the major and/or model fibrolytic bacteria to assess if they were represented in the current metagenomic dataset. At an E value of <1e-10, numerous genes in the present metagenomic dataset were related, but not identical or very similar, to the genes in any of the sequenced genomes (Table 2). Only one GH3 gene in the metagenomic dataset exhibited a high similarity (91% identity amino acid residues) to a GH3 gene of the genome of *P. ruminicola* 23, suggesting that none of the 120 fosmid clones was derived from the genomes of these species or closely related bacteria. The lack of sequences closely related to the genomes of these ‘major’ fibrolytic species is consistent with the finding of previous studies that used shotgun deep sequencing [3]-[5]. The CAZymes diversity and polysaccharide degrading capacity in the rumen is probably much larger than that represented by the known cellulolytic species.

**Table 2:** Number of ORFs in the present metagenomic dataset that exhibited similarities to the genes in the sequenced genomes of the rumen fibrolytic bacterial species.

Species	Total^a^	CAZy Genes						SP^h^	TM^i^	SP&TM^k^
		Total^b^	CBM^c^	GH^d^	GT^e^	CE^f^	PL^g^			
*Fibrobacter succinogenes* S85	1106	338	30	180	140	9	1	80	215	45
*Prevotella bryantii* B_1_4	1130	370	32	207	133	17	1	90	212	41
*Prevotella ruminicola* 23	1196	389	38	211 (1)*	137	29	1	99	215	43
*Ruminococcus albus* 7	1430	387	43	215	134	19	1	100	318	57
*Ruminococcus albus* 8	1475	377	35	210	135	19	1	103	358	66
*Ruminococcus flavefaciens* FD-1	1470	373	41	206	133	20	1	106	351	62

Notes: ^a^ total ORFs in the present metagenomic dataset that were found to be similar to the genes of each sequenced genome; ^b^ total ORFs in the metagenomic dataset that exhibited similarity to the CAZy genes of each sequenced genome; ^c-k^ ORFs in the metagenomic dataset that were found similar to the carbohydrate binding module (CBM), glycoside hydrolase (GH); glycosyl transferase (GT), carbohydrate esterase (CE), polysaccharide lyase (PL), signal peptide (SP), transmembrane domain (TM), and both SP and TM.

* One GH3 in the metagenomic dataset exhibited 91% amino acid similarity with a GH3 in the genome of *P*. *ruminicola* 23.

### Comparison with Other Metagenomic Datasets

The genes found in the present study were compared to those reported in the other large metagenomic datasets that were accessible [3], [5], [9]. About 84% of the 3,553 ORFs predicted in the present study were homologous (E value < 1e-10) to those reported of the Guernsey cow rumen [3]. Between these two datasets, only 129 ORFs (< 4% of the total genes identified in the present study) were identical, including 12 GH genes (including GH3, GH10, and GH43) and 14 other CAZy genes predicted in both studies. Besides, 875 ORFs (24.6% of the total ORFs of the present study) were > 90% similar to the ORFs predicted of Guernsey cow rumen, including 77 GH (e.g., GH3, GH5, GH9, GH10, and GH43) genes and 78 other CAZy genes. When compared to the two metagenomic datasets (fosmids and shotgun sequencing) of reindeer rumen [5], lower proportions of our predicted ORFs found very similar hits. Specifically, none of the ORFs identified in the present study shared > 87% similarity with those of the fosmid dataset and only 6% (214 genes in total) of our predicted ORFs were > 90% similar to those in the shotgun sequencing dataset of reindeer, including seven ORFs that were identical between the two datasets. None of these seven ORFs, however, was assigned to a CAZy family except one assigned to GT4. Of the remaining 207 predicted ORFs that were > 90% similar to the shotgun sequencing dataset of reindeer, only 14 were GH genes and 14 were other CAZy genes. The CAZy genes predicted in the present study were also compared to those of the foregut of wallaby dataset [9]. Although nearly 60% (2,342 in total) of the ORFs predicted in the present study appeared to be homologous (E value < 1e-10) to those of the wallabies, only 56 of them (< 1.6% of total) shared > 90% sequence similarity with their wallaby counterparts. Of these 56 predicted ORFs, 12 were identical to their wallaby counterparts. Among these 56 ORFs, seven were predicted to be CAZy genes. Only one GH3 and one GH4 were identical between the two datasets, but no β-1,4-endo-glucanase or xylanase were found to be highly similar (> 90%) between the two datasets. It was also noted that Sus-like PULs were found in all the sequenced metagenomes except for that derived from the gut of a wood-eating termite (Table S6, Figure 4).

The relative proportions of the major (Table 1) and all the (Table S7) predicted GH families also differed among the large metagenomes of rumen and herbivore gut. The comparisons led to two interesting observations. First, GH5 and GH9 were the most frequent cellulases found in the metagenomic studies. Second, the present dataset was enriched with GH3 (14.9% of total GHs), GH10 (10.8%), GH43 (8.7), GH5 (7.4%), and GH53 (5.6%). The large numbers of CAZy genes found in each of these metagenomic studies and yet the small proportion of the present CAZy genes that were highly similar to those reported of other ruminant species or breeds suggest that the rumen of the Jersey cows likely harbors a very different diversity of CAZymes. Although the differences might be partially attributable to the different techniques used (e.g., targeted sequencing of fosmid and BAC clones vs. shotgun metagenomic sequencing), differences in host genetics, feed, and feeding regimens might also be important factors that affect the diversity of CAZymes. This premise is consistent with the finding that the rumen microbiome can be significantly affected by feed and host genetics [14], [27]. Furthermore, proteins characteristic of cellulosomes (e.g., dockerins or scaffolding proteins), which are present in *R. flavefaciens* [28], were poorly represented in all the metagenomic datasets, including those produced by deep shotgun sequencing. Therefore, continued metagenomic research is needed to further explore and characterize the CAZymes diversity in the rumen of different cattle fed different diets.

## Materials and Methods

### Construction of the Metagenomic Fosmid Library

The animals were cared for following the guidelines for the Care and Use of Agricultural Animals in Agricultural Research and Teaching during the animal feeding and sample collection, which was approved by the Institutional Animal Care and Use Committee (IACUC) of The Ohio State University. The rumen digesta samples were collected from two Jersey cows fed mainly Timothy grass hay *ad libitum*. The solid fraction was separated from the liquid fraction, rinsed in an anaerobic buffer, and combined in equal portion as described previously [29]. Metagenomic DNA corresponding to the biofilm adherent to the fiber was extracted from the combined solid samples using the CTAB method [30]. DNA fragments ranging from 30 to 40 kb were isolated from the metagenomic DNA using sucrose ultracentrifugation [31] and then cloned into fosmids using the pCC1FOS fosmid library production kit (Epicentre Biotechnologies, Madison, WI).) per manufacturer’s protocol. All the recombinant colonies were picked and incubated in Luria-Bertani broth containing 12.5 µg/ml chloramphenicol at 37°C overnight. The fosmid clones were transferred to 384 well plates and stored at -80°C with a final DMSO concentration of 8%.

### Functional Screening

All clones were screened for polysaccharide digestion activity on selective plates supplemented with different substrates: 0.1% water soluble oat spelt xylan, 0.1% water soluble birchwood xylan [32], 0.1% carboxymethyl cellulose (CMC), or 1.0% potato starch as the sole carbon source. For β-glucosidase activity screening, esculin hydrate and ferric ammonium citrate were used as the substrate and indicator, respectively [33]. The screening plates were incubated at 37°C for up to 3 days for all the substrates except CMC. The CMC plates were incubated at 37°C for 2–4 days for colony growth and then at 30°C for another 3–7 days for CMCase activity expression. Reduced tryptone (0.15 – 0.25%) and yeast extract (0.075 – 0.125%) concentrations were also used to enhance CMCase induction.

To visualize β-glucosidase activity, the esculin and ferric plates were treated with chloroform vapor, and colonies that turned dark brown or black were identified as β-glucosidase positive [33]. The plates supplemented with xylan (soluble oat spelt or birchwood) or CMC were stained with 0.1% Congo Red followed by destaining with 1 M NaCl, and colonies with a clear surrounding halo were considered xylanase or cellulase positive [34]. The starch plates were stained by Gram’s iodine solution (0.2% I_2_ and 5.2% KI) and clones with a clear halo were recorded as positive.

### Pyrosequencing and *de novo* Assembly

The above activity screenings identified 120 positive clones in total. The fosmid DNA were extracted using a FosmidMAX DNA Purification Kit (Epicentre Biotechnologies). All the 120 fosmid DNA samples were pooled at equal amount and pyrosequenced by the Roche GS FLX system using Titanium chemistry. Individual reads were assembled first using Newbler (http://my454.com/products/analysis-software/), Mira [35], and Velvet [36] individually and the resulting contigs were then assembled again using Minimus 2 (http://amos.sourceforge.net). The final contigs were manually checked and potentially misassembled contigs were removed from the dataset. All the contigs sequenced were deposited in GenBank under accession numbers KC246771 - KC247082.

### Prediction and Analysis of Open Reading Frames

Opening reading frames (ORFs) were predicted from the assembled contigs using the GeneMark.hmm online server (http://exon.gatech.edu/GeneMark/metagenome/) with the refined Heuristic Models applied. The translated protein sequences of the predicted genes were subjected to Blastp comparison with the non-redundant protein database (NR, ftp://ftp.ncbi.nlm.nih.gov/blast/db/) and the CDD domain database (including Cluster of Ortholog Genes classification, COG, http://www.ncbi.nlm.nih.gov/cdd). Genes coding for CAZymes were identified by the CAZy Analysis Tool [37], [38]. The cutoff E value for the above analysis was set at <1e-5. The translated protein sequences were also compared to the predicted ORFs of complete or draft genome sequences (downloaded from GenBank) of individual ruminal species of fibrolytic bacteria, including *Fibrobacter succinogenes* S85 (GenBank accession number: NC_013410), *Ruminococcus albus* 7 (NC_014833), *Ruminococcus albus* 8 (NZ_ADKM00000000), *Ruminococcus flavefaciens* FD-1 (NZ_ACOK00000000), *Prevotella bryantii* B_1_4 (NZ_ADWO00000000), and *Prevotella ruminicola* 23 (NC_014033), using Blastp. The threshold of these comparisons was set to an E value of 1e-10. The predicted amino acid sequences were also compared to those produced from Guernsey cow rumen by shotgun metagenomic sequencing (http://portal.nersc.gov/project/jgimg/CowRumenRawData/submission/, using Blastp) [3], the fosmid dataset (http://genome.jgi.doe.gov/pages/dynamicOrganismDownload.jsf?organism=IMG_2199352020, using Blastp) and shotgun metagenomic dataset (http://genome.jgi.doe.gov/pages/dynamicOrganismDownload.jsf?organism=IMG_2081372005, using Blastp) of reindeer [5], and the assembled contig sequences of the wallaby foregut (GenBank accession number: ADGC00000000.1, using tBlastn) [9]. The cutoff E value was 1e-10 for all these comparisons. Signal peptide sequences were predicted using the SignalP 3.0 server (www.cbs.dtu.dk/services/SignalP/). Transmembrane domains were predicted using the TMHMM server 2.0 (http://www.cbs.dtu.dk/services/TMHMM/). The taxonomic assignment of the contigs was performed using PhyloPythiaS with a generic model [39] and MEGAN 4.0 [40] with the LCA-assignment algorithm (lowest common ancestor) implemented. A contig was assigned to a taxon if more than 50% of its predicted proteins showed the best Blastp match with a bacterium of that taxon. All the predicted ORFs were also subjected to taxonomic assignment by MEGAN. The ORFs were assigned to the lowest taxon, and the distribution of the assigned CAZy families among the lowest taxon (mostly genera) that had five or more predicted ORFs was analyzed. Metabolic pathways were reconstructed using the KEGG (http://www.kegg.jp/) implemented within MEGAN 4 [40].

## Supporting Information

Figure S1 **KEGG categories of the putative metabolic genes (A) and carbohydrate metabolism genes (B) identified in this study.**
(TIF)

Table S1 **Summary of the metagenomic dataset.**
(DOCX)

Table S2 **Taxonomic assignment of contigs longer than 10 kb by MEGAN and PhyloPythiaS.** CAZymes: The genes containing CAZy families. GH, glycoside hydrolase; GT, glycosyl transferase; CE, carbohydrate esterase; PL, polysaccharide lyase; SP, signal peptide; TM, transmembrane domain. *, one of the contigs is not assigned to Selenomonadales but to Firmicutes.
(XLSX)

Table S3 **Clusters of orthologous groups (COG) identified in the metagenomic dataset.**
(DOCX)

Table S4 **Putative polysaccharide utilization loci (PUL) discovered in the rumen metagenome.** * GenBank NR protein database. # Taxa assignment using MEGAN followed by PhyloPythiaS. The highlighted (red) PULs were the two Sus-like clusters described in the main text of the paper.
(XLSX)

Table S5 **Distribution of CAZy families identified in the microgenome.** * Predicted by MEGAN4 followed by PhyloPythiaS.
(XLSX)

Table S6 **The ORFs used in** **Figure 4** **.** * For Tammar Wallaby, reindeer and cow rumen, data were either downloaded from IMG database under each project or cited from paper [3], [5]. For Buffalo rumen fosmids, data were downloaded from Genbank (www.ncbi.nlm.nih.gov). For yak rumen, only four sequence information were available in Genbank at present and the rest information were cited from reference [7]. For this study, the Genbank accession numbers for the contigs were listed. † For tammar wallaby, buffalo rumen, reindeer and cow rumen, CAZy ID were adapted from the paper by Pope et al. [5]. For yak rumen, data were obtained from Genbank and the paper by Dai et al. [7]. # Sample ID (Acc. num.) were adapted from the paper by Pope et al. [5]. ‡ BACON, Bacteroidetes-Associated Carbohydrate-binding Often N-terminal.
(XLSX)

Table S7 **Comparison of all the GH families identified in large metagenomes of herbivores.** ^a^, Warnecke *et al.*, 2007. Metagenomic and functionalanalysis of hindgut microbiota of a wood-feeding higher termite. Nature 450:560-5. ^b^, Brulc, *et al.*, 2009. Gene centric metagenomics of the fiber-adherent bovine rumen microbiome reveals forage specific glycoside hydrolases. PNAS USA,106:1948-53. ^c^, Zhu *et al.*, 2011. Evidence of cellulose metabolism by the giant panda gut microbiome. PNAS USA, 108:17714-9. ^d^, Pope *et al.*, 2010. Adaptation to herbivory by the Tammar wallaby includes bacterial and glycoside hydrolase profiles different from other herbivores. PNAS USA,107:14793-8. ^e^, Dai *et al.*, 2012. Metagenomic insights into the fibrolytic microbiome in yak rumen. PLoS One 7(7):e40430. ^f^, Pope *et al.*, 2012. Metagenomics of the Svalbard reindeer rumen microbiome reveals abundance of polysaccharide utilization loci. PLoS One 7(6):e38571**.** ^g^, Hess *et al.*, 2011. Metagenomic discovery of biomass-degrading genes and genomes from cow rumen. Science, 468 331:463-7. NR, not reported. *, data from the BAC clones.
(XLSX)
