# Supplementary figures and images for: Metagenomic Insights into the Carbohydrate-Active Enzymes Carried by the Microorganisms Adhering to Solid Digesta in the Rumen of Cows
Source: PLoS One. 2013 Nov 5;8(11):e78507. doi: 10.1371/journal.pone.0078507 (PMC3818352; doi:10.1371/journal.pone.0078507)

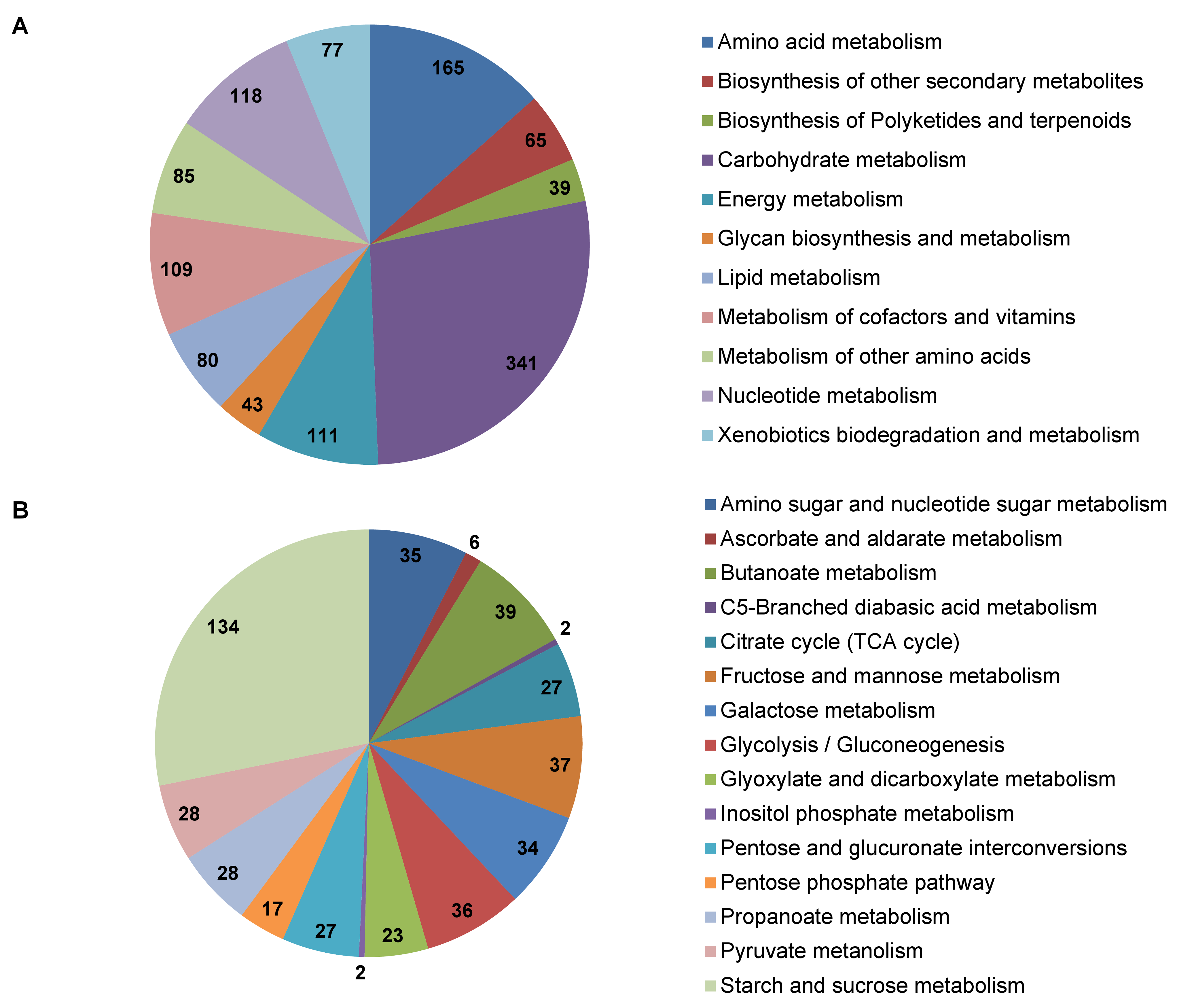

Supplement: Figure S1 — KEGG categories of the putative metabolic genes (A) and carbohydrate metabolism genes (B) identified in this study. (TIF) [file pone.0078507.s001.tif]
