# Supplementary material for: Metagenomic Insights into the Carbohydrate-Active Enzymes Carried by the Microorganisms Adhering to Solid Digesta in the Rumen of Cows
Source: PLoS One. 2013 Nov 5;8(11):e78507. doi: 10.1371/journal.pone.0078507 (PMC3818352; doi:10.1371/journal.pone.0078507)
Supplement: Table S1 — Summary of the metagenomic dataset. (DOCX) [file pone.0078507.s002.docx]

**Table S1.** Summary of the metagenomic dataset.

|  | **Number** |
| --- | --- |
| Total Reads | 616,494 |
| Average length (bp) of the sequencing reads | 455 |
| Total contigs | 1833 |
| Contigs with full-length insert | 66 |
| Contigs with length longer than 10kb | 41 |
| Contigs with length between 2kb to 10kb | 205 |
| Total ORFs predicted | 3,553 |
| ORFs from full-length insert contigs | 1,801 |
| ORFs from contigs of longer than 10kb but not full length | 969 |
| ORFs from contigs between 2kb to 10kb | 783 |
