# Supplementary material for: Metagenomic Insights into the Carbohydrate-Active Enzymes Carried by the Microorganisms Adhering to Solid Digesta in the Rumen of Cows
Source: PLoS One. 2013 Nov 5;8(11):e78507. doi: 10.1371/journal.pone.0078507 (PMC3818352; doi:10.1371/journal.pone.0078507)
Supplement: Table S3 — Clusters of orthologous groups (COG) identified in the metagenomic dataset. (DOCX) [file pone.0078507.s004.docx]

**Table S3**. Clusters of orthologous groups (COG) identified in the metagenomic dataset.

| **COG Category** | | **Code** | **Total** |
| --- | --- | --- | --- |
| **Information storage and processing** | |  |  |
|  | Translation, ribosomal structure and biogenesis | J | 140 (3.94%) |
|  | RNA processing and modification | A | 0 (0%) |
|  | Transcription | K | 188 (5.29%) |
|  | Replication, recombination and repair | L | 148 (4.16%) |
|  | Chromatin structure and dynamics | B | 0 (0%) |
| **Cellular processes and signaling** | | | |
|  | Cell cycle control, cell division, chromosome partitioning | D | 20 (0.56%) |
|  | Nuclear structure | Y | 0 (0%) |
|  | Defense mechanisms | V | 87 (2.45%) |
|  | Signal transduction mechanisms | T | 134 (3.77%) |
|  | Cell wall/membrane/envelope biogenesis | M | 125 (3.52%) |
|  | Cell motility | N | 17 (0.48%) |
|  | Cytoskeleton | Z | 0 (0%) |
|  | Extracellular structures | W | 0 (0%) |
|  | Intracellular trafficking, secretion, and vesicular transport | U | 33 (0.93%) |
|  | Posttranslational modification, protein turnover, chaperones | O | 65 (1.83%) |
| **Metabolism** | | | |
|  | Energy production and conversion | C | 132 (3.72%) |
|  | Carbohydrate transport and metabolism | G | 396 (11.1%) |
|  | Amino acid transport and metabolism | E | 219 (6.16%) |
|  | Nucleotide transport and metabolism | F | 124 (3.49%) |
|  | Coenzyme transport and metabolism | H | 123 (3.46%) |
|  | Lipid transport and metabolism | I | 62 (1.7%) |
|  | Inorganic ion transport and metabolism | P | 103 (2.90%) |
|  | Secondary metabolites biosynthesis, transport and catabolism | Q | 34 (0.96%) |
| **Poorly characterized** | | | |
|  | General function prediction only | R | 331 (12.78%) |
|  | Function unknown | S | 139 (5.47%) |
| **Total ORFs COG Annotated** | | | **2,423** |
